# Readability, reliability and quality of responses generated by ChatGPT, gemini, and perplexity for the most frequently asked questions about pain

**DOI:** 10.1097/MD.0000000000041780

**Published:** 2025-03-14

**Authors:** Erkan Ozduran, Ibrahim Akkoc, Sibel Büyükçoban, Yüksel Erkin, Volkan Hanci

**Affiliations:** a Sivas Numune Hospital, Physical Medicine and Rehabilitation, Pain Medicine, Sivas, Turkey; b University of Health Sciences, Basaksehir Cam and Sakura City Hospital, Anesthesiology and Reanimation, Istanbul, Turkey; c Dokuz Eylul University, Anesthesiology and Reanimation, Izmir, Turkey; d Dokuz Eylul University, Anesthesiology and Reanimation, Pain Medicine, Izmir, Turkey; e Dokuz Eylul University, Anesthesiology and Reanimation, Critical Care Medicine, Izmir, Turkey.

**Keywords:** artificial intelligence, ChatGPT, Gemini, online medical information, pain, perplexity

## Abstract

It is clear that artificial intelligence-based chatbots will be popular applications in the field of healthcare in the near future. It is known that more than 30% of the world’s population suffers from chronic pain and individuals try to access the health information they need through online platforms before applying to the hospital. This study aimed to examine the readability, reliability and quality of the responses given by 3 different artificial intelligence chatbots (ChatGPT, Gemini and Perplexity) to frequently asked questions about pain. In this study, the 25 most frequently used keywords related to pain were determined using Google Trend and asked to every 3 artificial intelligence chatbots. The readability of the response texts was determined by Flesch Reading Ease Score (FRES), Simple Measure of Gobbledygook, Gunning Fog and Flesch-Kincaid Grade Level readability scoring. Reliability assessment was determined by the Journal of American Medical Association (JAMA), DISCERN scales. Global Quality Score and Ensuring Quality Information for Patients (EQIP) score were used in quality assessment. As a result of Google Trend search, the first 3 keywords were determined as “back pain,” “stomach pain,” and “chest pain.” The readability of the answers given by all 3 artificial intelligence applications was determined to be higher than the recommended 6th grade readability level (*P* < .001). In the readability evaluation, the order from easy to difficult was determined as Google Gemini, ChatGPT and Perplexity. Higher GQS scores (*P* = .008) were detected in Gemini compared to other chatbots. Perplexity had higher JAMA, DISCERN and EQIP scores compared to other chatbots, respectively (*P* < .001, *P* < .001, *P* < .05). It has been determined that the answers given by ChatGPT, Gemini, and Perplexity to pain-related questions are difficult to read and their reliability and quality are low. It can be stated that these artificial intelligence chatbots cannot replace a comprehensive medical consultation. In artificial intelligence applications, it may be recommended to facilitate the readability of text content, create texts containing reliable references, and control them by a supervisory expert team.

## 1. Introduction

Pain affects all populations regardless of age, gender, income, race/ethnicity, or geography. Globally, it is estimated that 1 in 5 adults suffers from pain, and according to World Health Organization records, 1 in 10 adults are diagnosed with chronic pain each year.^[1]^ It is known that more than 30% of the world’s population lives with chronic pain, and that chronic pain has a significant impact on people’s lives by causing psychological disorders (e.g. depression and anxiety), sleep problems, loss of work capacity, economic difficulties and social withdrawal.^[2]^ Accessing health information via the internet is not a new method for patients. In studies conducted on individuals with chronic pain, access to pain-related information via the internet was found to be 24% of the population in one study and 39% in the other.^[3]^

Technology is moving one step further every day, and the internet is preferred in accessing medical information due to its convenience and cheapness.^[4]^ Not only this, medical literature is promoted and made available to patients through some social media platforms such as Google, Facebook, and Twitter.^[5]^ Today, a new technology called Artificial Intelligence (AI) has entered our lives, which aims to minimize human intervention and puts computer technology at the forefront and is being used in many areas of science, technology and health.^[6]^ AI; It refers to the development of computer systems that enable tasks that require human intelligence, such as making decisions, learning from experience, understanding natural language, recognizing images and solving problems. AI, which has the potential to revolutionize healthcare, has also made significant progress in the field of medicine. Artificial intelligence applied in the field of medicine consists of physical (smart prostheses for the disabled, elderly care, assistive robots during healthcare) and virtual (electronic health record system, neural network-based guidance) parts.^[7]^

Artificial intelligence-based chatbots and virtual assistants can interact with patients and answer their questions, provide basic medical information, and even help schedule appointments.^[8]^ As an example, version 3.5 of Chat Generative Pre-Trained Transformer (ChatGPT) was released in 2022 as a large language model (LLM) supported by artificial intelligence (AI), and a new alternative source of information emerged. ChatGPT has garnered a lot of attention for its ability to produce humane and coherent responses on a wide range of topics; It surpassed 100 million monthly active users in just 2 months and broke the record for the fastest growing app in history.^[9]^ Unlike the GPT-3.5 version, which is publicly available for free, the enhanced GPT-4 version is made available through a paid service.^[10]^ Developed as a rival to ChatGPT, Google Bard was developed in February 2023 and changed its name to Gemini in February 2024. The main difference between Gemini and ChatGPT is that Gemini is based on the language model for dialogue application (LaMDA) family, which consists of large language models.^[11]^ Google Gemini is capable of analyzing complex data sets such as images and subgraphs.^[12]^ Another chatbot, Perplexity, is based on OpenAI’s GPT technology and is specifically trained as a chatbot. Perplexity provides answers to prompts and questions and also contains links to quotes and related topics.^[13]^

The digital age has virtually transformed into an era in which patients receive information about their diseases and treatments via the internet and social media platforms with the help of patient education materials. However, patients can only read and understand this information according to their health literacy levels. National Institutes of Health (NIH), The American Medical Association and the United States Department of Health and Human Services have recommended that Internet-based patient education materials be written in a language below a sixth-grade level. Texts written above health literacy levels are considered difficult to read and understand by patients.^[14–17]^ In addition, it has been observed that the answers generated by artificial intelligence robots raise concerns about the accuracy and reliability of these answers, as they may evoke non-existent sources and produce biased and incorrect information.^[18]^ It has been stated that these artificial intelligence applications produce responses based on well-known patterns and structures without reasoning or thinking functions like humans.^[19]^ For this reason, the readability, reliability and quality of the answers given by artificial intelligence chatbots are questionable due to their effects on human health.

In light of the above-mentioned information, this study aimed to examine the reliability, quality and readability of the answers given by 3 different artificial intelligence chatbots (ChatGPT, Gemini and Perplexity) to frequently asked questions about pain. To our knowledge, this study is the first research in this field and aims to contribute to the existing literature in this field and set an example for future studies.

## 2. Materials and methods

### 2.1. Ethical approval

This cross-sectional study was prepared after receiving ethics committee approval (Cumhuriyet University Ethics Committee, Ethics committee No: 2024/05-26, Date: May 16, 2024).

### 2.2. Research protocol

Before the internet search, all data from personal browsers was deleted. The research was conducted by logging out of Google accounts and turning on Google Incognito mode. The most frequently searched keywords related to pain were tried to be reached through Google Trends (https://trends.google.com/) on May 16, 2024.^[20,21]^ Health subheadings from all over the world, from 2004 to the present, were selected as search criteria. In the results section, the “most relevant” questions were selected. As a result of Google web searches, the 25 most frequently searched keywords covering various categories were recorded, and geographical areas of interest were recorded according to sub-regions.

The resulting keywords were asked separately in English to 3 chatbots [ChatGPT-3.5 (Open AI), Google Gemini (Google AI) and Perplexity (Perplexity AI)], which are publicly available free of charge.^[6,10,22]^ As the conversations continued, the answers given for each keyword could be affected by the previous keywords and answers, so each keyword was entered by different users and answers were tried to be obtained.

Unlike the GPT-3.5 version, which is publicly available free of charge, the GPT-4 version is accessed after a paid subscription. In our study, instead of the current GPT-4 version, GPT-3.5 version, which can be accessed by people with low socioeconomic levels, was used. In our study, artificial intelligence applications that were freely accessible were used.^[6,10,22]^

### 2.3. Reliability assessment

The reliability of the answers obtained was determined based on The Journal of the American Medical Association (JAMA) Benchmark criteria. The following 4 points are evaluated in the JAMA criteria: disclosure, authorship, attribution, and currency. In this evaluation, each variable is given a score of 0 or 1, resulting in a final score between 0 and 4. A low JAMA score represents low reliability, and a high score represents high reliability.^[23]^

The Modified DISCERN scale is another reliability scale used in our study. This scale consists of 5 criteria. If the criterion is met, it receives 1 point; if it does not exist, it receives 0 points. The final score ranges from 0 to 5, with higher scores representing higher reliability. The questions included in the scale are: “Are additional sources of information listed for patient reference?,” “Are reliable sources of information used?,” “Does the text address areas of uncertainty?,” “Is the information provided balanced and unbiased??,” and “Are the aims clear and understandable?.”^[24]^ The validity and reliability of the JAMA and DISCERN scales have been evaluated in the literature.^[23,25]^

### 2.4. Quality assessment

Global Quality Score (GQS) is a scale in which each criterion used to evaluate the quality of online resources is worth 1 point and a maximum of 5 points can be obtained. The scale rating is as follows: 1 point: poor quality, not at all useful to patients, 2 points: overall poor quality for very limited use by patients, 3 points: fair quality, somewhat useful to patients, 4 points: good quality, useful to patients, 5 points: excellent quality, very useful for patients.^[26]^

The Ensuring Quality Information for Patients (EQIP) tool was another quality indicator used in our study. It is a scale that indicates not only the quality of writing but also the clarity of information. The 20 questions in the scale are answered as “yes,” “partly” “no” or “does not apply.” 1 point is given for a yes answer, 0.5 for a partly answer, and 0 for a no answer. The points obtained are added up and divided by 20, then those that do not apply are subtracted and multiplied by 100 [((# of Yes*1) + (# of Partly*0.5) + (# of No*0))/20 − (# of does not apply)] *100 = % score.^[27]^ According to the EQIP results, results between 76% and 100% are “well written,” results between 51% and 75% are “good quality with minor problems,” results between “26% and 50%” are considered “serious problems with quality,” and those between “0%–25%” are expressed as “severe problems with quality.”^[28]^ Validity and reliability evaluations have been made in the literature for the GQS and EQIP surveys.^[23,25]^

### 2.5. Readability assessment

All responses received from artificial intelligence chatbots were evaluated using 2 different internet websites with readability calculation features. Responses from all 3 AI applications were evaluated using both http://readabilityformulas.com/ (Calculator 1) and https://www.online-utility.org/english/readability_test_and_improve.jsp (Calculator 2). Flesch Kincaid Grade Level (FKGL), The Flesch Reading Ease Score (FRES), Gunning Fog Readability (GFOG), Simple Measure of Gobbledygook (SMOG), Automated Readability Index (ARI), Coleman-Liau Readability Index (CLI) and Linsear Write The readability of the relevant texts was calculated using the (LW) readability formula.^[10,22,26]^ Calculation explanations of the formulas are given in detail in Table 1. The readability scores of the answers given by all 3 artificial intelligences to the determined questions were noted as median (minimum-maximum). The accepted readability level score is 80.0 for FRES and 6 for the other 6 formulas.^[6]^

**Table 1:** Readability tools, formulas and descriptions

Readability index	Description	Formula
Flesch Reading Ease Score(FRES)	It was created to assess the readability of newspapers and is particularly effective for evaluating school textbooks and technical manuals. This standardized test is utilized by numerous US government agencies. The scores range from 0 to 100, with higher scores indicating greater ease of reading.	I = (206.835 − (84.6 X (B/W)) − (1.015 X (W/S)))
Gunning FOG (GFOG)	It was designed to assist American businesses in enhancing the readability of their written content and is applicable across various disciplines. It estimates the number of years of education required for a person to understand a given text.	G = 0.4 X (W/S+((C*/W) X 100))
Simple Measure of Gobbledygook (SMOG)	It is typically appropriate for middle-aged readers, ranging from 4th grade to college level. While it aims to test 100% comprehension, most formulas measure about 50%-75% comprehension. It is most accurate when applied to documents that are at least 30 sentences long. It measures the number of years of education the average person needs to understand a text.	G = 1.0430 X √ C + 3.1291
Flesch–Kincaid grade level (FKGL)	Part of the Kincaid Navy Personnel test collection, it was designed for technical documentation and is suitable for a wide range of disciplines. Delineates the academic capacity level imperative for grasping the written material	G = (11.8 X (B/W)) + (0.39 X (W/S)) − 15.59
Automated readability index (ARI)	The ARI (Automated Readability Index) has been utilized by the military for writing technical manuals. Its calculation provides the grade level required to comprehend the text. Assesses the scholastic rank in American educational institutions needed to be capable of comprehending written material. The greater the number of characters, the more complex the term.	ARI = 4.71 X l + 0.5*ASL − 21.43
Coleman–Liau (CL) score	It is designed for middle-aged readers, spanning from 4th grade to college level. The formula is based on text with a grade level range of 0.4 to 16.3 and is applicable to many industries. Evaluates the educational level required for understanding a text and offers an associated grade level in the US education system.	G = (−27.4004 X (E/100)) + 23.06395
Linsear Write (LW)	It was developed for the United States Air Force to assist in calculating the readability of their technical manuals. Offers an approximate assessment of the academic level needed to comprehend the text.	LW = (*R* + 3C)/S Result • If > 20, divide by 2 • If ≤ 20, subtract 2, and then divide by 2

ASL = the average number of sentences per 100 words, B = number of syllables, C = complex words (≥3 syllables), C* = complex words with exceptions including, proper nouns, words made 3 syllables by addition of “ed” or “es,” compound words made of simpler words, E = predicted Cloze percentage = 141.8401 − (0.214590 × number of characters) + (1.079812 * S), G = grade level, I = Flesch Index Score, R = the number of words ≤ 2 syllables, S = number of sentences, SMOG = Simple Measure of Gobbledygook, W = number of words.

The readability of the responses generated by artificial intelligence was evaluated by 2 independent senior researchers in the field of pain (E.Ö. and V.H.). In addition, the quality and reliability of the answers given were recorded separately by the same 2 researchers, and the results obtained from both were noted by taking their average.

### 2.6. Statistical analysis

Statistical analysis was conducted using SPSS Windows 24.0 (SPSS Inc., Chicago). Frequency data are presented as numbers (n) and percentages (%), while continuous data are shown as medians (minimum-maximum). The Chi-square test and Fisher’s exact test were employed to compare frequency variables, and the Mann–Whitney *U* test and Wilcoxon tests were used to compare continuous data. To assess the consistency of the calculators, intraclass correlation coefficient (ICC) analysis was performed for each formula. A *P* value of <.05 was considered to indicate a significant difference.

## 3. Results

**Figure 1.**
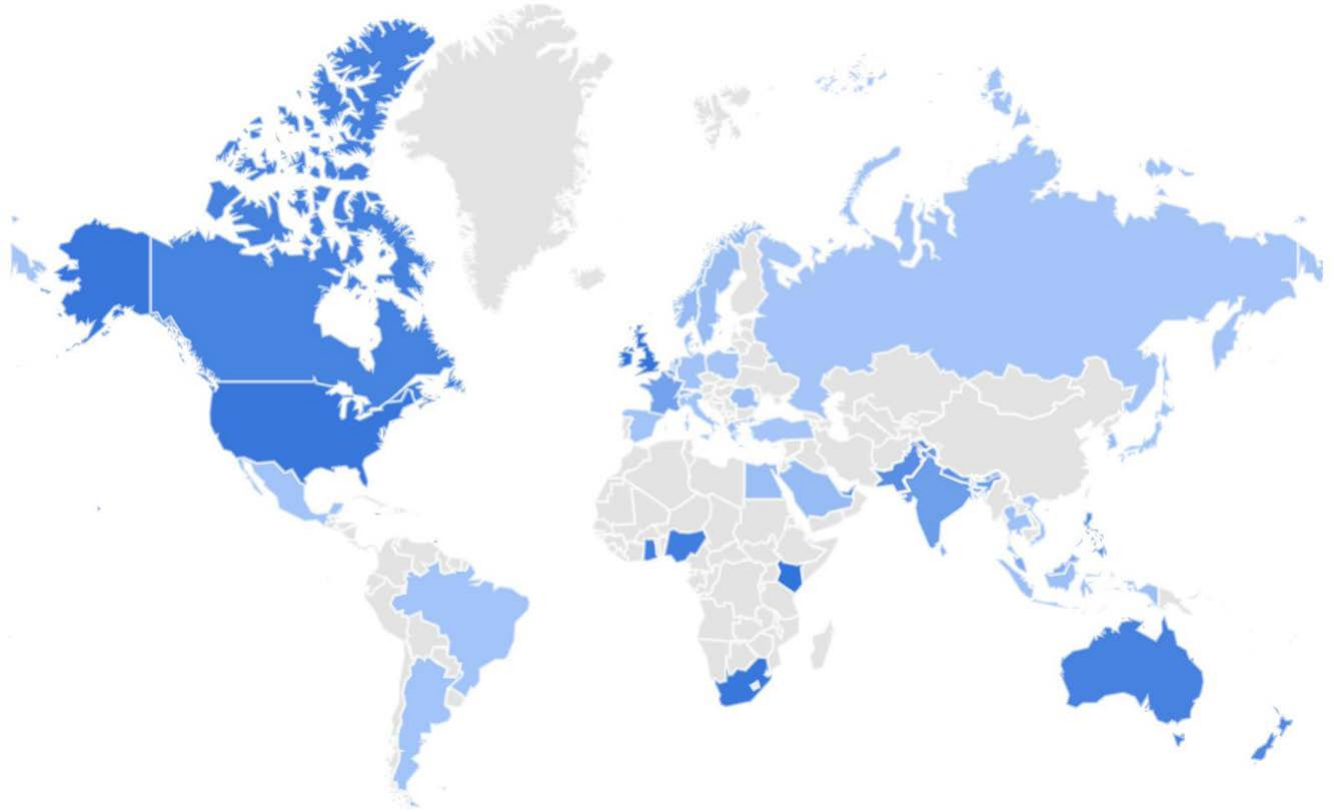
Search interest in pain across the countries: 2004–2023 (*Data source:* Google Trends, https://www.google.com/trends).

As a result of Google Trend search, the first 3 keywords were determined as “back pain,” “stomach pain,” and “chest pain.” Since there was already a keyword called “Abdominal pain” before “Abdomen pain,” it was removed from the analysis. “Pain in Stomach” was removed from the analysis because there was already a keyword called “Stomach pain.” “Pain in Chest” was removed from the analysis because there was already a keyword called “Chest pain.” Therefore, the study was conducted on 22 keywords. The complete list of keywords is presented in Table 2. Jamaica, Trinidad and Tobago and Kenya were identified as the 3 countries with the highest pain-related searches, respectively. Search interest for the keyword pain across countries is shown in Figure 1. Using Calculator 1 and 2, the readability scores of the answers given by ChatGPT-3.5, Google Gemini and Perplexity programs to the most frequently asked questions about pain were determined. The readability scores of the texts were analyzed by comparing them with the 6th grade reading level. Tables 3 to 6 show the results.

**Table 2:** Top 22 relevant keywords searched about pain across countries: 2004–2023 (based on google trends data)

Rank	Keyword	Category of the topic based on EQIP
1	Back Pain	Condition or illness
2	Stomach Pain	Condition or illness
3	Chest Pain	Condition or illness
4	Lower Back Pain	Condition or illness
5	Knee Pain	Condition or illness
6	Neck Pain	Condition or illness
7	Shoulder Pain	Condition or illness
8	Left Side Pain	Condition or illness
9	Abdominal Pain	Condition or illness
10	Leg Pain	Condition or illness
11	Right Side Pain	Condition or illness
12	Hip Pain	Condition or illness
13	Foot Pain	Condition or illness
14	Muscle Pain	Condition or illness
15	Joint Pain	Condition or illness
16	Nerve Pain	Condition or illness
17	Ear Pain	Condition or illness
18	Breast Pain	Condition or illness
19	Kidney Pain	Condition or illness
20	Period Pain	Condition or illness
21	Pain Management	Discharge or aftercare
22	Heart Pain	Condition or illness

EQIP = ensuring quality information for patients.

**Table 3:** Readability scores for ChatGPT-3.5, Gemini, and Perplexity responses to the most frequently asked pain-related questions, and a statistical comparison of the text content to a 6th-grade reading level [median (minimum-maximum)], using Calculator 1. (https://readabilityformulas.com/free-readability-formula-tests.php)

Calculator 1 statistics	ChatGPT 3.5	Google Gemini	Perplexity	Chat GPT C6thGRL (*P*)*†	Gemini C6thGRL (*P*)*†	Perplexity C6thGRL (*P*)*†	Between Chatgpt and Gemini‡	Between Chatgpt and perplexity‡	Between gemini and perplexity‡
FRES	31.50 (9–55)	41.50 (22–55)	34 (10–70)	**<.001**	**<.001**	**<.001**	**.005**	.860	**.026**
GFOG	17.65 (13.60–25.80)	15.75 (12–25.09)	20.45 (15.60–54.60)	**<.001**	**<.001**	**<.001**	**.007**	**.001**	**<.001**
FKGL	14.19 (10.74–20.92)	12.23 (7.79–15.99)	16.99 (13.13–49.57)	**<.001**	**<.001**	**<.001**	**.001**	**.001**	**<.001**
CLI	15.17 (11.37–21.52)	13.61 (11.08–16.28)	15.77 (12.31–18.63)	**<.001**	**<.001**	**<.001**	**.008**	.213	**<.001**
SMOG	12.81 (9.77–18.09)	11.57 (8.67–16.42)	14.68 (11.57–32.37)	**<.001**	**<.001**	**<.001**	**.005**	**.002**	**<.001**
ARI	16.32 (12.58–23.50)	14.37 (10.14–26.16)	19.71 (14.15–60.62)	**<.001**	**<.001**	**<.001**	**.015**	**.001**	**<.001**
LW	16.37 (12.34–22.28)	14.22 (7.42–31.10)	21.41 (14.50–8*5*.25)	**<.001**	**<.001**	**<.001**	**.019**	**<.001**	**<.001**
Grade level	15.00 (12.00–21.00)	13.00 (10.00–20.00)	17.00 (13.00–45.00)	**<.001**	**<.001**	**<.001**	**.020**	**.002**	**<.001**
Reading level	n (%)	n (%)	n (%)						
Difficult to read	1 (4.5)	5 (22.7)	0 (0)				**<.001**	**<.001**	**<.001**
Very difficult to read	3 (13.6)	6 (27.3)	1 (4.5)						
Extremally difficult to read	13 (59.1)	7 (31.8)	19 (86.4)						
Professional	5 (22.7)	3 (13.6)	2 (9.1)						
Somewhat difficult	0 (0)	1 (4.5)	0 (0)						
Readers age	n (%)	n (%)	n (%)						
8–9 years old (Fourth and Fifth graders)	0 (0)	1 (4.5)	0 (0)				**.019**	.255	**<.001**
10–11 years old (Fifth and Sixth graders)	0 (0)	0 (0)	0 (0)						
11–13 years old (Sixth and Seventh graders)	0 (0)	0 (0)	0 (0)						
12–14 years old (Seventh and Eighth graders)	0 (0)	0 (0)	0 (0)						
13–15 years old (Eighth and Ninth graders)	0 (0)	0 (0)	0 (0)						
14–15 years old (Ninth to Tenth graders)	0 (0)	0 (0)	0 (0)						
15–17 years old (Tenth to Eleventh graders) n (%)	0 (0)	1 (4.5)	0 (0)						
17–18 years old (Twelfth graders)	1 (4.5)	4 (18.2)	0 (0)						
18–19 years old (college level entry)	3 (13.6)	6 (27.3)	1 (4.5)						
21–22 years old (college level)	5 (22.7)	3 (13.6)	2 (9.1)						
23+ years old	13 (59.1)	7 (31.8)	16 (72.7)						
College graduate	0 (0)	0 (0)	3 (13.6)						

*P* values in bold are statistically significant.

ARI = Automated Readability Index, CLI = Coleman-Liau Index, FKGL = Flesch-Kincaid Grade Level, FRES = Flesch reading ease score, GFPG = Gunning FOG, LW = Linsear Write, SMOG = Simple Measure of Gobbledygook.

* C6thGRL (*P*): Comparison of the responses according to 6th grade reading level (*P*).

† Wilcoxon test.

‡ Chi-Square test for categorical variables and Mann–Whitney *U* test for continuous variables.

**Table 4:** Readability scores for ChatGPT-3.5, Gemini, and Perplexity responses to the most frequently asked questions about pain, along with a statistical comparison of text content to a 6th-grade reading level [median (minimum-maximum)], using Calculator 2. (https://www.online-utility.org/english/readability_test_and_improve.jsp)

CALCULATOR 2 Statistics	ChatGPT	Gemini	Perplexity	ChatGPT C6thGRL (*P*)*†	Gemini C6thGRL (*P*)*†	Perplexity C6thGRL (*P*)*†	Between ChatGPT and Gemini (*P*)‡	Between ChatGPT and Perplexity (*P*)‡	Between perplexity and gemini (*P*)‡
FRES	29.83 (9.59–51.29)	41.17 (25.26–53.63)	33.16 (9.83–72.16)	**<.001**	**<.001**	**<.001**	**.004**	.647	**.023**
GFOG	16.43 (13.12–25.16)	13.91 (10.71 –21.09)	19.04 (14.85–51.96)	**<.001**	**<.001**	**<.001**	**.002**	**.004**	**<.001**
FKGL	13.98 (11.25–20.04)	11.99 (7.41–16.14)	16.40 (13.18–49.91)	**<.001**	**<.001**	**<.001**	**.001**	**.003**	**<.001**
CLI	14.93 (11.43–22.30)	13.36 (11.02–16.06)	15.76 (12.34–18.71)	**<.001**	**<.001**	**<.001**	**.003**	.130	**<.001**
SMOG	15.31 (12.70–19.46)	13.37 (11.35–16.22)	16.90 (14.49–32.75)	**<.001**	**<.001**	**<.001**	**.001**	**.003**	**<.001**
ARI	15.14 (11.71–20.94)	13.23 (10.11–21.68)	17.80 (13.16–59.59)	**<.001**	**<.001**	**<.001**	**.008**	**.004**	**<.001**

*P* values in bold are statistically significant.

ARI = Automated Readability Index, CLI = Coleman-Liau Index, FKGL = Flesch-Kincaid Grade Level, FRES = Flesch reading ease score, GFPG = Gunning FOG, LW = Linsear Write, SMOG = Simple Measure of Gobbledygook.

* C6thGRL (*P*): Comparison of the responses according to 6th grade reading level (*P*).

† Wilcoxon test.

‡ Chi-Square test for categorical variables and Mann–Whitney *U* test for continuous variables.

**Table 5:** Analyzing the readability metrics of ChatGPT-3.5, Gemini, and Perplexity responses to the 22 most frequently asked questions concerning pain, and conducting a statistical evaluation of text complexity against a 6th-grade reading standard [median (minimum-maximum)], utilizing the average values derived from Calculator 1 and Calculator 2 for comparison

Readability indexes	ChatGPT*	ChatGPT C6thGRL (*P*)†‡	Gemini*	Gemini C6thGRL (*P*)†‡	Perplexity*	Perplexity C6thGRL (*P*)†‡	Between ChatGPT and gemini§	Between ChatGPT and perplexity (*P*)§	Between perplexity and gemini (*P*)§
FRES	30.49 (9.30–53.15)	**<.001**	41.72 (24.13–53.32)	**<.001**	32.22 (10.58–71.08).	**<.001**	**.005**	.805	**.019**
GFOG	17.14 (13.36–24.48)	**<.001**	14.81 (12.21–23.09)	**<.001**	19.83 (15.38–53.28)	**<.001**	**..003**	**.002**	**<.001**
FKGL	14.17 (11–20.48)	**<.001**	12.16 (7.71–15.82)	**<.001**	16.71 (13.16–49.74)	**<.001**	**.001**	**.003**	**<.001**
SMOG	14.10 (11.24– 18.77)	**<.001**	12.35 (10.61–15.83)	**<.001**	15.86 (13.17–32.56)	**<.001**	**.002**	**.002**	**<.001**
CLI	14.82 (10.83– 22.41)	**<.001**	13.39 (11.05–16.01)	**<.001**	15.74 (12.33–18.67)	**<.001**	**.016**	.108	**<.001**
ARI	15.68 (12.22–22.22)	**<.001**	13.78 (11.06–23.92)	**<.001**	18.76 (13.66–60.26)	**<.001**	**.008**	**.002**	**<.001**

Calculator 1: https://readabilityformulas.com/free-readability-formula-tests.php

Calculator 2: https://www.online-utility.org/english/readability_test_and_improve.jsp

*P* values in bold are statistically significant.

ARI = Automated Readability Index, CLI = Coleman-Liau Index, FKGL = Flesch-Kincaid Grade Level, FRES = Flesch reading ease score, GFPG = Gunning FOG, LW = Linsear Write, SMOG = Simple Measure of Gobbledygook.

* [(Calculator 1) + (Calculator 2)]/2.

† C6thGRL (*P***):** Comparison of the responses according to 6th grade reading level (*P***).**

‡ Wilcoxon test.

§ Chi-Square test for categorical variables and Mann–Whitney *U* test for continuous variables.

**Table 6:** Comparison of JAMA, EQIP, Modified DISCERN and Global Quality Scale (GQS) ratings for the responses from ChatGPT-3.5, Gemini, and Perplexity

	ChatGPT vs Perplexity			ChatGPT vs Gemini			Perplexity vs Gemini		
	ChatGPT	Perplexity	*P*	ChatGPT	Gemini	*P*	Perplexity	Gemini	*P*
GQS, n (%)			.247*			**.008** *			**.008** *
1-point	0 (0)	0 (0)		0 (0)	0 (0)		0 (0)	0 (0)	
2-point	6 (27.3)	2 (9.1)		6 (27.3)	1 (4.5)		2 (9.1)	1 (4.5)	
3-point	8 (36.4)	12 (54.5)		8 (36.4)	3 (13.6)		12 (54.5)	3 (13.6)	
4-point	8 (36.4)	8 (36.4)		8 (36.4)	18 (81.8)		8 (36.4)	18 (81.8)	
5-point	0 (0)	0 (0)		0 (0)	0 (0)		0 (0)	0 (0)	
JAMA, n (%)			**<.001** *			.312*			**<.001** *
0-point	22 (100)	0 (0)		22 (100)	21 (95.5)		0 (0)	21 (95.5)	
1-point	0 (0)	0 (0)		0 (0)	0 (0)		0 (0)	0 (0)	
2-point	0 (0)	2 (9.1)		0 (0)	0 (0)		2 (9.1)	0 (0)	
3-point	0 (0)	18 (81.8)		0 (0)	1 (4.5)		18 (81.8)	1 (4.5)	
4-point	0 (0)	2 (9.1)		0 (0)	0 (0)		2 (9.1)	0 (0)	
m DISCERN, n (%)			**<.001** *			.167*			**<.001** *
1-point	2 (9.1)	0 (0)		2 (9.1)	0 (0)		0 (0)	0 (0)	
2-point	9 (40.9)	0 (0)		9 (40.9)	5 (22.7)		0 (0)	5 (22.7)	
3-point	11 (50)	3 (13.6)		11 (50)	16 (72.7)		3 (13.6)	16 (72.7)	
4-point	0 (0)	18 (81.8)		0 (0)	1 (4.5)		18 (81.8)	1 (4.5)	
5-point	0 (0)	1 (4.5)		0 (0)	0 (0)		1 (4.5)	0 (0)	
EQIP, n (%)			**.002** *			**.023** *			**.009** *
Serious problems with good quality	8 (36.4)	2 (9.1)		8 (36.4)	1 (4.5)		2 (9.1)	1 (4.5)	
Good quality with minor problems	14 (63.6)	11 (50)		14 (63.6)	20 (90.9)		11 (50)	20 (90.9)	
Well written	0 (0)	9 (40.9)		0 (0)	1 (4.5)		9 (40.9)	1 (4.5)	

*P* values in bold are statistically significant.

EQIP = ensuring quality information for patients.

* Chi-Square test.

### 3.1. ChatGPT-3.5 analysis outcomes utilizing average results from Calculator 1 and 2

During the text readability examination of 22 ChatGPT-3.5 responses generated in reaction to 22 questions, the median GFOG was 17.14 (13.36–24.48) and median FRES was 30.49 (9.30–53.15). Furthermore, the median SMOG and FKGL were 14.10 (11.24–18.77) and 14.17 (11–20.48) years of education, respectively. ARI was 15.68 (12.22–22.22) and CLI was 14.82 (10.83–22.41) years of education (Table 5).

### 3.2. Gemini analysis outcomes utilizing average results from Calculator 1 and 2

During the text readability examination of Gemini’s 22 responses the median GFOG was 14.81 (12.21–23.09) and the median FRES was 41.72 (24.13–53.32). Furthermore, the median SMOG, FKGL, ARI, and CLI means were 12.35 (10.61–15.83), 12.16 (7.71–15.82), 13.78 (11.06–23.92) and 13.39 (11.05–16.01) years of education, respectively (Table 5).

### 3.3. Perplexity analysis outcomes utilizing average results from Calculator 1 and 2

During the text readability examination of 22 Perplexity responses, the median GFOG was 19.83 (15.38–53.28) and the median FRES was 32.22 (10.58–71.08). Furthermore the median SMOG and FKGL were 15.86 (13.17–32.56) and 16.71 (13.16–49.74) years of education, respectively. ARI was 18.76 (13.66–60.26) years of education and CLI was 15.74 (12.33–18.67) years of education (Table 5).

### 3.4. Assessment of readability across the 3 groups, utilizing average scores from Calculators 1 and 2

When assessing the readability of responses among all 3 groups by averaging the outcomes from Calculator 1 and 2, significant differences emerged between specific groups. Significant differences were observed between: ChatGPT-3.5 and Gemini groups (*P* < .001), ChatGPT-3.5 and Perplexity groups (*P* < .001), as well as Perplexity and Gemini groups (*P* < .001). Moreover, no significant differences were found in the comparison between ChatGPT and Perplexity groups regarding CLI and FRES, respectively (*P* = .108, *P* = .805) (Table 5).

Based on the readability assessments, all readability metrics, excluding FRES, are arranged in a hierarchy of readability from easiest to most difficult: Google Gemini, ChatGPT-3.5, and Perplexity. Nonetheless, as per the FRES readability metric, the order varies slightly: Google Gemini, Perplexity, and ChatGPT-3.5 (Table 5).

### 3.5. Assessing ChatGPT, Gemini, and Perplexity responses based on the suggested reading level for sixth graders

Upon comparing the median readability scores of all responses with the sixth-grade reading standard, a statistically significant difference was noted for all metrics in contrast to the sixth-grade level (*P* < .001). Notably, when compared with all metrics, the readability of responses surpassed the sixth-grade benchmark. Statistically significant findings were also obtained when comparing Calculator 1 and 2 results, and also the average of both calculators (*P* < .001) (Tables 3–5).

### 3.6. Reliability and quality assessment

The JAMA, DISCERN, GQS, and EQIP evaluation results (mean (minimum-maximum)) of the answers given by Google Gemini were as follows; 0 (0–3), 3 (2–4), 4 (2–4) and 64.29 (42.86–80). The JAMA, DISCERN, GQS, and EQIP evaluation results (mean (minimum–maximum)) of ChatGPT’s answers were as follows; 0 (0–0), 2.5 (1–3), 3 (2–4), 57.14 (28.57–71.43). The JAMA, DISCERN, GQS, and EQIP evaluation results (mean (minimum–maximum)) of the answers given by Perplexity were as follows; 3 (2–4), 4 (3–5), 3 (2–4) and 71.43 (33.30–85.71).

Gemini’s answers were found to have the highest GQS scores (*P* = .008). Perplexity’s answers achieved the top JAMA, modified DISCERN and EQIP scores, respectively (*P* < .001, *P* < .001, *P* < .05) (Table 6).

### 3.7. Intraclass correlation coefficients (ICCE)

GFOG, FRES, CL, FKGL, ARI, and SMOG scores were computed using 2 different calculators (https://www.online-utility.org/english/readability_test_and_improve.jsp, https://readabilityformulas.com/free-readability-formula-tests.php).

### 3.8. ICCE for Chat GPT

The intraclass correlation coefficient for FRES was 0.981, for KFGL was 0.953, for GFOG was 0.923, for CL was 0.990, for ARI was 0.922 and for SMOG was 0.940.

### 3.9. ICCE for gemini

The intraclass correlation coefficient for FRES was 0.945, for KFGL was 0.879, for GFOG was 0.940, for CL was 0.922, for ARI was 0.927 and for SMOG was 0.864.

### 3.10. ICCE for perplexity

The intraclass correlation coefficient for FRES was 0.922, for KFGL was 0.991, for GFOG was 0.990, for CL was 0.999, for ARI was 0.991 and for SMOG was 0.990.

### 3.11. ICCE for GQS, JAMA, and mDISCERN

The intraclass correlation coefficients were 0.837 for GQS, 0.980 for JAMA, 0.819 for mDISCERN 0.914 for EQIP at Gemini.

The intraclass correlation coefficients were 0.931 for GQS, 0.805 for mDISCERN 0.931 for EQIP at ChatGPT.

The intraclass correlation coefficients were 0.886. for GQS, 0.761 for JAMA, 0.750 for mDISCERN 0.970 for EQIP at Perplexity.

## 4. Discussion

In this study, the readability, quality and reliability of the responses given by Gemini, Perplexity and ChatGPT artificial intelligence applications to the most frequently asked questions about pain were evaluated. Responses from all 3 AI applications were found to have reading levels higher than the recommended 6th-grade reading level. To our knowledge, this study represents the first and pioneering research effort on this topic, focusing specifically on responses generated by 3 different popular AI chatbots to frequently asked questions related to pain and evaluating information reliability, quality, and readability levels.

Readability is a definition that expresses how easily a text can be understood. It is stated that the average adult’s reading level in the USA is at the 7th to 8th grade level. It is estimated that the number of people with low literacy skills in the USA is 43 million. It has been reported that 8.4 million of these people are illiterate, that is, they lack the reading and writing skills needed for many activities and life.^[29]^ For this reason, it is clear that writing texts in a way that individuals can easily read them will increase understandability. Despite this, it has been reported in the literature that many online patient education materials have higher than recommended readability levels, and this situation is worrisome for public health.^[15–17,26]^

Studies in the literature evaluating online information about pain have shown that readability levels are higher than recommended. In the study, in which 71 Google and 15 government-based websites were evaluated, chronic pain was questioned and the readability level was determined to be “fairly difficult to read” and was suitable for classes between the ages of 15 and 17 or between the ages of 10 and 12. Again, it was reported that less than 30% of the websites evaluated in the same study met all JAMA criteria, so there were concerns about reliability. The study emphasized that the availability of barely readable text on online platforms is a significant barrier to providing health information that can help people struggling with chronic pain.^[30]^ Artificial intelligence can reportedly help healthcare professionals diagnose diseases, plan treatments, predict outcomes, and manage public health. In addition, it has been reported that it may affect patients’ decision-making processes regarding healthcare services.^[31]^ Unlike other study methodologies, we tried to shed light on the readability, quality and reliability evaluation of the responses given by artificial intelligence robots, which is a popular method today, to the most frequently asked questions about pain, rather than websites.

There are a limited number of studies in the literature on the readability, quality and reliability of ChatGPT, Gemini and Perplexity artificial intelligence chatbots on various medical subjects. In their study on carpal tunnel release, Croen et al^[32]^ found that ChatGPT’s answers were longer than Google’s answers and that the readability of ChatGPT answers was statistically significantly more difficult than Google’s answers. On a positive note, contrary to their difficulties in readability, ChatGPT chatbots have always advised their users to “contact their surgeon” in case of relevant inquiries. In their study on ChatGPT regarding frequently asked questions about Surgical Treatment of Retinal Diseases, Momenaei et al^[33]^ found that the answers given to questions about macular holes, common vitreoretinal surgeries for retinal detachments and epiretinal membranes were appropriate, but difficult or very difficult to read for the average ordinary person. They stated that it was very difficult and that college graduation would be required to understand the text.^[33]^ Kim et al^[34]^ asked 5 questions to ChatGPT, Bard and Microsoft Bing Chat regarding the “Definition and Differentiation of Supportive Care, Palliative Care and Hospice Care” and determined the readability level of the answers received as 10th grade level. Finally, in their study, they emphasized that patients and healthcare professionals should be careful when using these platforms as information sources.^[34]^ Gül et al^[6]^ reported that in the answers to 100 questions about subdural hematoma asked to ChatGPT, Bard, and perplexity, readability levels were higher than the recommended 6th grade level. They emphasized that although these artificial intelligence robots have the possibility of improving health outcomes and patient satisfaction, efforts should be made to ensure that the responses provided are at the appropriate level of readability. Haver et al^[35]^ in their study of 25 questions on ChatGPT, 3.5, GPT-4, and Bard regarding lung cancer and lung cancer imaging, found that the overall average readability of the answers given by all 3 artificial intelligence chatbots was very difficult for the average adult patient (>8. grade level). It has been stated that BARD is easier to read and readable than its competitors, and that the answers are clinically appropriate. Şahin et al^[36]^ in their study investigating the answers given by 5 artificial intelligence chatbots (ChatGPT, Bard, Bing, Ernie, and Copilot) to questions about erectile dysfunction, found that the general readability levels were high. In the subgroup analysis, it was emphasized that BARD was the easiest to read and ChatGPT required a high level of education to understand. Finally, they emphasized that by using algorithms combined with human control, the results produced can be reconstructed to meet average readability standards. Lee et al^[37]^ examined the answers given by ChatGPT and Gemini to the American College of Cardiology’s 52 frequently asked questions on hypertension education and found that readability levels were at the collegiate level, but both chatbots responded with a high degree of accuracy. They emphasized that although Gemini’s responses consist of more words, ChatGPT responses have significantly higher readability levels than Gemini.

In our study, it was determined that the answers given to the most frequently asked pain questions by all 3 artificial intelligence chatbots had readability levels higher than the recommended 6th grade level. Gemini showed the easiest readability, followed by ChatGPT and Perplexity. Our study results are similar to other studies in the literature. It is clear that artificial intelligence chatbots providing readable answers that an adult can easily understand will have positive effects on health literacy and public health.

Another striking issue in our study was the reliability and quality evaluation of the responses received. Musheyev et al^[38]^ in their study where they analyzed the answers given by ChatGPT, Perplexity, Chat Sonic, and Microsoft Bing AI chatbots to questions about urological malignancies, they determined that the answers given were moderate to high information quality (median DISCERN score 4 out of 5, range 2–5). Cocci et al^[39]^ obtained low quality results in their study on ChatGPT responses for urology patients. In their study where Gül et al^[6]^ examined the answers to 100 questions about subdural hematoma asked to ChatGPT, Bard, and perplexity, they found low JAMA and DISCERN scores in ChatGPT and Gemini. On the other hand, it was emphasized that these scores were found to be significantly higher in Perplexity. In our study, similar to the literature, low JAMA, modified DISCERN scores were detected in Gemini and ChatGPT, while significantly higher EQIP, JAMA and modified DISCERN scores were detected in Perplexity. The fact that Perplexity answers the questions asked with its sources and that the information presented in these sources includes the authors and current dates has resulted in high quality and reliability scores. By providing artificial intelligence chatbots with a broader and more accurate information database on health-related issues, more reliable and quality answers can be produced. During the development of artificial intelligence models such as ChatGPT, the use of surveys that ensure the quality and reliability of online information may be the subject of developments that will positively affect public health in future updated versions. However, although access to health via the internet is increasing day by day, it cannot replace a proper medical evaluation and examination by health professionals. It is not possible to obtain the medical evaluation required for correct diagnosis and treatment online.^[40]^ In addition, there are concerns about privacy violations, data security, and that the quality of medical information shared through these platforms is not guaranteed. These concerns cause potential risks to occur in important decision-making processes.^[41]^

### 4.1. Strength of the study

In our study, we used multiple popular artificial intelligence chatbots, not just one as in many other study methodologies, and investigated the relationship between them. In addition, we tried to standardize the readability evaluation by using not one but 2 different calculators that are publicly available. We also tried to examine the compatibility between calculators.

### 4.2. Limitations of the study

Our study has some limitations. Our study, which was created using only the 22 most popular keywords, may have somewhat limited the analysis related to pain. Future analysis with more keywords will shed light on the literature. In our study, we analyzed the most trending English keywords. Research in languages other than English will also reveal how the subject is in other languages. In our study, we analyzed only ChatGPT, Gemini and Perplexity chatbots due to their popularity and ease of use and accessibility. Studies to be conducted with other chatbots will shed light on the readability, reliability and quality of artificial intelligence applications. In our study, only the responses given by chatbots dated May 16, 2024 were evaluated. Therefore, it is clear that different answers given by chatbots on a different date will change the study results.

## 5. Conclusion

Our study attempted to evaluate the readability, reliability and quality of pain-related content created by ChatGPT, Gemini and Perplexity artificial intelligence chatbots. The readability levels of all 3 chatbots were determined to be higher than the recommended 6th grade readability level. It has been determined that Perplexity offers reliable and quality content on pain compared to other artificial intelligence chatbots. In order for artificial intelligence chatbots to transfer information beneficial to public health in the future, they need to expand their databases, produce content with reliable academic references, and supervise the content created by creating a team of experienced experts on the subject. Although such technological developments seem to facilitate access to some health-related information, it is obvious that they cannot replace face-to-face medical consultation between doctor and patient.

## Author contributions

**Conceptualization:** Erkan Ozduran, Yüksel Erkin, Volkan Hanci.

**Data curation:** Erkan Ozduran, Ibrahim Akkoc, Sibel Büyükçoban, Yüksel Erkin, Volkan Hanci.

**Formal analysis:** Erkan Ozduran, Ibrahim Akkoc, Sibel Büyükçoban, Yüksel Erkin, Volkan Hanci.

**Investigation:** Erkan Ozduran, Ibrahim Akkoc, Sibel Büyükçoban.

**Methodology:** Erkan Ozduran, Ibrahim Akkoc, Sibel Büyükçoban, Volkan Hanci.

**Project administration:** Erkan Ozduran, Volkan Hanci.

**Resources:** Erkan Ozduran, Sibel Büyükçoban.

**Supervision:** Erkan Ozduran, Ibrahim Akkoc, Sibel Büyükçoban, Yüksel Erkin, Volkan Hanci.

**Validation:** Erkan Ozduran.

**Visualization:** Erkan Ozduran.

**Writing – original draft:** Erkan Ozduran, Ibrahim Akkoc, Sibel Büyükçoban, Yüksel Erkin, Volkan Hanci.

**Writing – review & editing:** Ibrahim Akkoc, Sibel Büyükçoban, Yüksel Erkin, Volkan Hanci.
